# Urinary Microvesicle-Bound Uromodulin: A Potential Molecular Biomarker in Diabetic Kidney Disease

**DOI:** 10.1155/2017/3918681

**Published:** 2017-01-15

**Authors:** Neng-jun Lou, Yi-hong Ni, Hong-ying Jia, Jing-ti Deng, Lu Jiang, Feng-jie Zheng, Ai-li Sun

**Affiliations:** ^1^The Second Hospital of Shandong University, 247 Beiyuan Street, Ji'nan, Shandong 250033, China; ^2^Department of Biochemistry, School of Medicine of Shandong University, Shandong, China

## Abstract

This study was designed to investigate the changes of urinary microvesicle-bound uromodulin and total urinary uromodulin levels in human urine and the correlations with the severity of diabetic kidney disease (DKD). 31 healthy subjects without diabetes and 100 patients with type 2 diabetes mellitus (T2DM) were included in this study. The patients with T2DM were divided into three groups based on the urinary albumin/creatinine ratio (UACR): normoalbuminuria group (DM, *n* = 46); microalbuminuria group (DN1, *n* = 32); and macroalbuminuria group (DN2, *n* = 22). We use a specific monoclonal antibody AD-1 to capture the urinary microvesicles. Urinary microvesicle-bound uromodulin and total urinary uromodulin levels were determined by enzyme-linked immunosorbent assay (ELISA). Our results showed that the levels of urinary microvesicle-bound uromodulin in DN1 and DN2 groups were significantly higher than those in control group and DM group (*P* < 0.01). Multiple stepwise linear regression analysis showed that UACR was independent determinant for urinary microvesicle-bound uromodulin (*P* < 0.05) but not for total urinary uromodulin. These findings suggest that the levels of urinary microvesicle-bound uromodulin are associated with the severity of DKD. The uromodulin in urinary microvesicles may be a specific marker of DKD and potentially may be used to predict the onset and/or monitor the progression of DKD.

## 1. Introduction

Diabetic kidney disease (DKD) is a major complication of diabetes mellitus and the most frequent cause of end-stage renal disease (ESRD) [1, 2]. Early diagnosis and treatment have become an important issue in clinical work. Traditionally, incipient nephropathy is defined by the appearance of microalbuminuria [3], but it does not correlate well with underlying glomerular damage, since diabetic subjects with microalbuminuria display tremendous heterogeneity when concomitant biopsies are examined [4]. Therefore, new specific indicators of DKD might be useful to monitor the disease progression or regression and are required to accurately target these patients for therapeutic intervention earlier in the course of the disease. Uromodulin is a GPI-anchored glycoprotein and produced by the thick ascending limb of the loop of Henle of the mammalian kidney. It is present in large aggregates of up to several million Da in urine physiologically [5], and the monomeric molecule has a molecular weight of about 85 kDa. Uromodulin excretion in urine follows proteolytic cleavage of the ectodomain of its glycophosphatidy linositol-anchored counterpart that is situated on the luminal cell surface of the loop of Henle. The levels of uromodulin had marked change in urinary excretion in pathological conditions [5–7]. Therefore, uromodulin was considered useful as a marker of renal disease [8].

It has been found that many cells can produce microvesicles, including hematopoietic cells, reticulocytes, B/T lymphocytes, dendritic cells, mast cells, platelets, intestinal epithelial cells, astrocytes, neurons, fetal cells, and tumor cells [9–11]. So far, the material composition, biological properties, and function of the microvesicles are mostly based on the in vitro experiments of cultured cell lines or animal models. In recent years, people began to pay attention to the state of the microvesicles in the complex physiological environment, the study that the microvesicles in body fluid have gradually become a hot topic. Microvesicles in human serum, urine, follicular center, semen, prostatic fluid, amniotic fluid, and malignant pleural effusion and ascites are widely distributed, suggesting that they may play an irreplaceable role in physiological and pathological conditions.

Urinary microvesicles, including microparticles and exosomes, have recently been the targets of urine proteomic analysis [12–14]. Urinary microparticles with a size range between 100 and 1000 nm are membrane-shed vesicles, while exosomes with a diameter of 30–100 nm are membrane vesicles secreted by tubular cells. They all have the same lipid bilayer, but the majority of microvesicles isolated from urine are thought to be exosomes. They may mediate a variety of physiological and pathological functions. The microvesicles exist in many body fluids, such as blood and urine. Collection of urine is simple and noninvasive so it is one of the most useful resources. These microvesicles can be used as a new molecular biomarker for renal dysfunction and structural damage [15].

Urinary microvesicles are derived from the kidney and contain proteins secreted by the kidney tissue mostly. Based on this, several recent articles have reported new potential markers of kidney disease in urinary microvesicles [16–18].

We hypothesised that uromodulin might be a protein component of urinary microvesicles, and the microvesicle-bound uromodulin represents kidney underlying protein alterations. To confirm this hypothesis, we have developed an enzyme-linked immunosorbent assay (ELISA) method for urinary microvesicles-bound uromodulin using a specific monoclonal antibody, AD-1, developed by Deng et al. [19, 20]. After it is immobilized on the plate, the antibody can specifically capture and purify the intact microvesicles from urine samples, and the uromodulin activity in the urinary microvesicles can be determined. With this assay, levels of urinary microvesicle-bound uromodulin in patients with T2DM were tested and compared with age- and sex-matched normal individuals. We pooled all 24 h urine samples from a subject and then used a portion of that for our analysis. The applicability of urinary microvesicle-bound uromodulin as an early biomarker for determining the status of DKD was evaluated and the levels of uromodulin in microvesicles and whole urine samples were directly compared.

## 2. Materials and Methods

### 2.1. Subject Selection

In this study, 31 age- and sex-matched Chinese Han nondiabetic healthy subjects and 100 patients with type 2 diabetes mellitus (T2DM) were recruited from the Second Hospital of Shandong University. The patients with T2DM were divided into three groups based on the urinary albumin/creatinine ratio (UACR): normoalbuminuria group (DM, *n* = 46, ACR < 30 mg/g creatinine); microalbuminuria group (DN1, *n* = 32, ACR from 30–300 mg/g creatinine); and macroalbuminuria group (DN2, *n* = 22, ACR > 300 mg/g creatinine). There were no significant differences in the three groups with respect to age and sex. In addition, 31 control subjects were enrolled after a careful history and clinical examination with informed consent, and protocols were approved by the institutional ethics committees.

All patients with hypertension or proteinuria were treated with angiotensin converting enzyme inhibitors (ACEI) and/or angiotensin receptor blockers (ARB), patients with diabetes were treated with human insulin or repaglinide, and patients with hypercholesterolemia were treated with 3-hydroxy-3-methylglutaryl-coenzyme A (HMG-CoA) reductase inhibitors. No additional medicines were taken within 1 week of urine collection. Exclusion criteria were as follows: tumors, urinary tract disorders, pregnancy, known renal diseases other than DKD, decompensated heart failure, chronic inflammatory diseases, prostatic diseases (in males), hematologic diseases, liver diseases, and recent myocardial infarction or unstable angina within the past 6 months.

### 2.2. Urinary and Serum Samples Collection

Urine samples were collected from all subjects with a 24 h period. Two 0.95 ml aliquots of the urine sample were collected and neutralized with 50 *μ*l aliquots of neutralization buffer (1 M Tris-HCl, pH 7.4, containing 1% NaN3). The samples were immediately stored at −80°C until analysis. After 10 hours, overnight fasting blood samples of patients with T2DM and healthy individuals were collected by venipuncture. The serum samples were collected and stored at −80°C until analysis after centrifugation at 400*g* for 10 min.

### 2.3. Biochemical Parameters Assays

The following methods were used to assay the biochemical parameters in the serum samples: liquid chromatography for glycosylated hemoglobin (HbA1c), chemical modification method for triglyceride (TG), cholesterol (CH), low density lipoprotein cholesterol (LDL-C) and high density lipoprotein cholesterol (HDL-C), Jaffe-kinetic assay for creatinine (CR), and urease-GLDH method for blood urea nitrogen (BUN).

### 2.4. Preparation of the Specific Monoclonal Antibody to the Microvesicle (AD-1) Plate

The microvesicle specific monoclonal antibody (AD-1) was initially obtained from the serum of liver cancer patients with anti-human membrane associated liver alkaline phosphatase [21], to culture the (lymphocyte) hybrid tumor cells in RPMI 1640 containing 10% fetal bovine serum and to collect the culture supernatant containing AD-1 antibody. The monoclonal antibody against human uromodulin was obtained commercially from R&D Systems.

### 2.5. AD-1 Plate Preparation

Use the tris buffer solution (10 mmol/L Tris, 10 mmol/L NaCl, and 10 mmol/L NaN3, pH 8.5) to dilute the concentration of goat anti-mouse antibody to 10 ug/ml. Distribute the diluted antibody to the 96-well plate (Costar Co. Ltd., USA, 50 *μ*l/well) at 37°C to make the plate dry and then wash the plate with phosphate buffered saline containing 0.05% Tween-20 (PBST) once. The 50 mmol/L Tris/HCl (including 150 mmol/L NaCl) buffer solution was used to dilute the antibody concentration to 20 *μ*g/ml. Add 50 *μ*l of culture supernatant containing the AD-1 antibody to each well and put the plate at 4°C overnight. The coated plate was washed again with PBST and incubated at room temperature for 1 h. Then, it was stored at 4°C with 200 *μ*l/well of incubation buffer (50 mmol/l TrisHCl, pH 8.0, containing 0.15 mol/l NaCl and 0.05% NaN3) to be used later [16].

### 2.6. Determination of the Total Urinary Uromodulin and Urinary Microvesicle-Bound Uromodulin

Total urinary uromodulin was determined by a sandwich ELISA kit (R&D System). The verification of the specificity of the ELISA method has been proven in our previous article [16]. Urinary microvesicle-bound uromodulin was separated by a filtra-centrifugation method. Urine samples (500 *μ*l) were centrifuged at 17,000*g* for 10 min at 4°C and removed from the urinary sediment, to transfer the supernatant into filtration concentrators (100 kDa MWCO, Millipore) and centrifuge it at 4000*g* for 5–10 min under normal temperature to let almost all solution go through the filters. After washing the filters with PBS, PBS containing 0.1% Triton-X100 was added in the concentrators and incubated on ice for 5 min with agitation to dissolve the urinary microvesicle-bound uromodulin. This step was repeated with 50 *μ*l of PBS-Triton buffer. A total of 150 *μ*l of solution containing urinary microvesicle-bound uromodulin was collected. We added 50 *μ*l of urine samples to the AD-1 coated plate, per well, covered with an adhesive strip and incubated 2 h at room temperature. After washing the plate with PBST three times, 50 *μ*l of rabbit anti-human uromodulin antibody was added to each well and incubated for another 2 h at room temperature. 50 *μ*l of the working dilution of Streptavidin-HRP (R&D System) was added to each well after washing the plate with PBST three times and incubated for another 2 h at room temperature. Avoid placing the plate in direct light. Substrate solution was added to each well after three washes with PBST and incubated for 20 min at room temperature. Also avoid placing the plate in direct light. Add 25 *μ*l of stop solution to each well and gentle tap the plate to ensure thorough mixing. Determine the optical density of each well immediately, using a microplate reader set to 450 nm.

### 2.7. Statistical Analyses

Measurement data, which met normal distribution, were presented as mean ± SEM, and comparisons were tested statistically using one-way analysis of variance followed by the appropriate post hoc test for determining statistical significance among various groups. Urinary microvesicle-bound uromodulin and total urinary uromodulin, which did not meet normal distribution, were presented as median (*P*_25_, *P*_75_) and comparisons were tested statistically using Kruskal-Wallis *H* test. Multiple stepwise linear regressions and Pearson correlation were used to analyze the relationship of urinary microvesicle-bound uromodulin with total urinary uromodulin, HbA1c, UACR, CR, BUN, CH, TG, LDL-C, HDL-C, and blood pressure. All data were two-tailed, analyzed by SPSS software 21.0 (SPSS, Chicago, IL, USA) and *P* values <0.05 were considered significant.

## 3. Results

### 3.1. Clinical Characteristics Analysis

The anthropometric and biochemical characteristics of baseline are shown in Table 1. We can see that there are no significant differences in the age and gender among each group. The HbA1c of DM, DN1, and DN2 groups was significantly higher than the healthy control group (*P* < 0.05), while the differences among the three diabetic groups were not statistically significant. DM, DN1, and DN2 groups had increased UACR (*P* < 0.05) compared with the control group and significant differences among themselves. DN2 group had significantly elevated LDL-C compared with other groups, while the levels of HbA1c were not significantly different in three diabetic groups (Table 1).

### 3.2. Excretion of Urinary Microvesicles-Bound Uromodulin and Total Urinary Uromodulin

The levels of urinary microvesicle-bound uromodulin in DN1 and DN2 groups were significantly higher than those in control group and DM group (*P* < 0.01). The levels of urinary microvesicle-bound uromodulin in DN2 group were significantly higher than that in DN1 group (*P* < 0.01). The levels of total urinary uromodulin in DM and DN1 groups were significantly higher than that in control group (*P* < 0.01) and in DN2 group they were significantly lower than DN1 group (Table 2).

### 3.3. Correlation and Multiple Linear Regression Analysis

Correlation analysis showed that the levels of urinary microvesicle-bound uromodulin were positively correlated with UACR, HbA1c, systolic blood pressure, and total urinary uromodulin (*P* < 0.05) (Table 3). Multiple stepwise linear regression analysis showed that UACR and HbA1c were independent determinants for urinary microvesicle-bound uromodulin (*P* < 0.05) but not for total urinary uromodulin (Table 4).

## 4. Discussion

The cellular and molecular mechanisms that lead to DKD are not completely identified. It is known that the renal functional changes are associated with cellular and extracellular derangements in both the glomerular and tubulointerstitial compartments [22], which could be reflected by urinary microvesicles. By mass spectrometry analysis, it was found that the urinary microvesicles containing 295 proteins were associated with renal and systemic diseases [23]. Therefore, it is helpful to find out the significance of the analysis of the microvesicles related proteins in normal and pathological conditions [24].

The purification technology of the microvesicles is mainly supercentrifugation and filtration. Because the supercentrifugation needs expensive equipment and a very long period of time, we use the filter instead of the supercentrifugation. However, the filtration did not separate the microvesicles that the diameter less than 300 nm [25]. We successfully captured and purified the uromodulin in urinary microvesicles in urine samples first by using our specific monoclonal antibody AD-1 and ELISA. This method has merit including rapid and high repetitiveness and has high sensitivity and specificity [21].

In our study, the excretion of total urinary uromodulin in the T2DM patients without albuminuria and with microalbuminuria was significantly increased compared with healthy subjects. It is consistent with previous studies that uromodulin expression in the early stages of DKD increased in the T2DM patients [26–28]. We consider that the excretion of total urinary uromodulin increases when there is damage to ascending Henle's loop of the tubules and may be associated with renal hyperfiltration. The excretion of total urinary uromodulin in the T2DM patients with macroalbuminuria was significantly decreased compared with those with microalbuminuria. It is consistent with other studies that focused on the later stages and suggested decreased uromodulin expression in DKD [8, 29]. The excretion of total urinary uromodulin may decrease as a result of reduction in renal mass and indicates dysfunction of the renal medulla.

AD-1 staining is only present on the brush border of proximal tubular cells in the cortex (S1 and S2 segments) and in the outer strip of the outer medulla and in the medullary rays (S3 segments) [16]. So the microvesicle-bound uromodulin in urine may be secreted from tubular epithelial cells and be related to early tubular impairment. Our data showed that the urinary microvesicle-bound uromodulin excretion was obviously increased in DN1 and DN2 groups, especially in the DN2 group, but was at a low level in the control group and DM group. The changes of urinary microvesicle-bound uromodulin were in concordance with the alterations of UACR in healthy subjects and the three groups of patients with T2DM. The level of urinary microvesicle-bound uromodulin in DM group was higher than the control group (no statistical significance was found may be due to the small sample size). In future study, we should expand the sample size and observe the glomerular filtration rate (GFR). We assess the predictive potential for GFR in combination with UACR and urinary microvesicle-bound uromodulin, thus allowing a more precise staging of the disease and individualization of therapy.

The increased excretion of urinary microvesicle-bound uromodulin was correlated with the UACR, HbA1c, systolic blood pressure, and total urinary uromodulin. In a further multiple stepwise linear regression analysis, UACR and HbA1c were independent determinants for urinary microvesicle-bound uromodulin. This result suggested that the urinary microvesicle-bound uromodulin may be a specific marker of DKD.

Recent study has shown that some patients with diabetes have advanced renal pathological changes and progressive kidney function decline even if urinary albumin levels are in the normal range, indicating that albuminuria is not the perfect marker for the early detection of DKD [30]. The measurement of urinary albumin remains the gold standard for diagnosing and categorizing DKD, so the sensitivity and specificity of the marker need to be compared with urinary albumin. The present article provides a relevant biomarker (microvesicle-bound uromodulin) that has been found to be associated with DKD.

This study has limitations such as small number of subjects, wide distributions for age, no GFR, different duration of diabetes, and effect of drugs. Further improvements are needed in our future study. However, all these findings indicate that urinary microvesicle-bound uromodulin is a specific marker for DKD and potentially may be used to predict the onset and/or monitor the progression of DKD.

## Figures and Tables

**Table 1:** Baseline anthropometric and biochemical characteristics.

Parameter	Control group	DM group	DN1 group	DN2 group
Number (*n*)	31	46	32	22
Age (year)	56.5 ± 7.85	55.02 ± 11.41	52.79 ± 15.12	51.56 ± 17.09
Gender (male/female)	15/16	28/18	13/19	12/10
UACR (mg/g)	5.75 ± 2.63	9.8 ± 4.38	91.6 ± 46.44^ab^	642.79 ± 332.86^abc^
HbA1c (%)	4.62 ± 0.43	8.45 ± 2.72^a^	8.98 ± 2.56^a^	9.63 ± 2.3^a^
CR (mmol/l)	51.13 ± 13.17	61.78 ± 15.21	70.58 ± 21.19	95.03 ± 46.11
BUN (mmol/l)	4.33 ± 0.84	4.21 ± 0.99	6.75 ± 2.97	6.03 ± 2.41
Systolic blood pressure (mmHg)	139.5 ± 12.66	142.68 ± 20.82	138.7 ± 14.8	162.57 ± 19.97
Diastolic blood pressure (mmHg)	79.25 ± 10.78	82.47 ± 9.87	81.5 ± 12.66	83.43 ± 9.62
CH (mmol/l)	4.83 ± 0.92	4.9 ± 1.62	4.13 ± 1.3	5.58 ± 2.3
TG (mmol/l)	2.03 ± 0.95	1.68 ± 0.94	1.78 ± 0.43	1.53 ± 0.76
LDL-C (mmol/l)	1.78 ± 0.4	2.76 ± 0.74	2.21 ± 0.8	2.22 ± 0.53^a^
HDL-C (mmol/l)	1.24 ± 0.57	1.3 ± 0.3	1.08 ± 0.25	1.09 ± 0.3

^a^*P* < 0.05 versus control group; ^b^*P* < 0.05 versus DM group; ^c^*P* < 0.05 versus DN1 group.

DM = type 2 diabetes mellitus with normoalbuminuria; DN1 = type 2 diabetes mellitus with microalbuminuria; DN2 = type 2 diabetes mellitus with macroalbuminuria; UACR = urinary albumin/creatinine ratio; HbA1c = glycosylated hemoglobin; TG = triglyceride; CH = cholesterol; LDL-C = low density lipoprotein cholesterol; HDL-C = high density lipoprotein cholesterol; CR = creatinine; BUN = blood urea nitrogen.

**Table 2:** The changes of urinary microvesicle-bound uromodulin and total urinary uromodulin.

Group	*n*	Urinary microvesicle-bound uromodulin (pg/ml)	Total urinary uromodulin (pg/ml)
Control	31	0.251 (0.233, 0.277)	0.469 (0.330, 1.309)
DM	46	0.262 (0.247, 0.569)	1.083 (0.719, 1.349)^a^
DN1	31	0.298 (0.279, 0.311)^ab^	1.320 (1.022, 1.457)^a^
DN2	19	0.337 (0.272, 0.352)^abc^	1.047 (0.319, 1.453)^c^

^a^*P* < 0.01 versus control group; ^b^*P* < 0.01 versus DM group; ^c^*P* < 0.01 versus DN1 group.

**Table 3:** The correlation analysis among urinary microvesicle-bound uromodulin and total urinary uromodulin and variables (*P* value).

Variables	Urinary microvesicle-bound uromodulin
UACR	0.002
HbA1c	0.034
Systolic blood pressure	0.049
Total urinary uromodulin	0.000

**Table 4:** The stepwise multiple linear regression analysis among urinary microvesicle-bound uromodulin and total urinary uromodulin and variables (*P* value).

Variables	Urinary microvesicle-bound uromodulin
UACR	0.000
HbA1c	0.046
Systolic blood pressure	0.200
Total urinary uromodulin	0.064

## References

[1] Reutens A. T. (2013). Epidemiology of diabetic kidney disease. *Medical Clinics of North America*.

[2] Park C. W. (2014). Diabetic kidney disease: from epidemiology to clinical perspectives. *Diabetes and Metabolism Journal*.

[3] Remuzzi G., Schieppati A., Ruggenenti P. (2002). Nephropathy in patients with type 2 diabetes. *New England Journal of Medicine*.

[4] Fioretto P., Mauer M. (2007). Histopathology of diabetic nephropathy. *Seminars in Nephrology*.

[5] Lau W.-H., Leong W.-S., Ismail Z., Gam L.-H. (2008). Qualification and application of an ELISA for the determination of tamm horsfall protein (THP) in human urine and its use for screening of kidney stone disease. *International Journal of Biological Sciences*.

[6] Sejdiu I., Torffvit O. (2008). Decreased urinary concentration of Tamm-Horsfall protein is associated with development of renal failure and cardiovascular death within 20 years in type 1 but not in type 2 diabetic patients. *Scandinavian Journal of Urology and Nephrology*.

[7] Akioka Y., Chikamoto H., Horita S. (2009). Screening of vesicoureteral reflux in pediatric patients with kidney transplantation showing non-specific interstitial fibrosis and tubular atrophy with interstitial Tamm-Horsfall protein deposits in protocol allograft biopsy. *Clinical Transplantation*.

[8] Möllsten A., Torffvit O. (2010). Tamm-Horsfall protein gene is associated with distal tubular dysfunction in patients with type 1 diabetes. *Scandinavian Journal of Urology and Nephrology*.

[9] De Broe M. E., Roels F., Nouwen E. J., Claeys L., Wieme R. J. (1985). Liver plasma membrane: the source of high molecular weight alkaline phosphatase in human serum. *Hepatology*.

[10] Lin X. P., Almqvist N., Telemo E. (2005). Human small intestinal epithelial cells constitutively express the key elements for antigen processing and the production of exosomes. *Blood Cells, Molecules, and Diseases*.

[11] Fauré J., Lachenal G., Court M. (2006). Exosomes are released by cultured cortical neurones. *Molecular and Cellular Neuroscience*.

[12] Wang Y., Chen L.-M., Liu M.-L. (2014). Microvesicles and diabetic complications—novel mediators, potential biomarkers and therapeutic targets. *Acta Pharmacologica Sinica*.

[13] Raimondo F., Corbetta S., Morosi L. (2013). Urinary exosomes and diabetic nephropathy: a proteomic approach. *Molecular BioSystems*.

[14] Zubiri I., Posada-Ayala M., Benito-Martin A. (2015). Kidney tissue proteomics reveals regucalcin downregulation in response to diabetic nephropathy with reflection in urinary exosomes. *Translational Research*.

[15] Musante L., Tataruch D. E., Holthofer H. (2014). Use and isolation of urinary exosomes as biomarkers for diabetic nephropathy. *Frontiers in Endocrinology*.

[16] Sun A.-L., Deng J.-T., Guan G.-J. (2012). Dipeptidyl peptidase-IV is a potential molecular biomarker in diabetic kidney disease. *Diabetes and Vascular Disease Research*.

[17] Kalani A., Mohan A., Godbole M. M. (2013). Wilm's tumor-1 protein levels in urinary exosomes from diabetic patients with or without proteinuria. *PLoS ONE*.

[18] Zhou H., Kajiyama H., Tsuji T. (2013). Urinary exosomal Wilms' tumor-1 as a potential biomarker for podocyte injury. *American Journal of Physiology—Renal Physiology*.

[19] Deng J. T., Hoylaerts M. F., Nouwen E. J., De Broe M. E., Van Hoof V. O. (1996). Purification of circulating liver plasma membrane fragments using a monoclonal antileucine aminopeptidase antibody. *Hepatology*.

[20] Ti Deng J., Parsons P. G. (1988). Solid phase immunoassay for high molecular weight alkaline phosphatase in human sera using a specific monoclonal antibody. *Clinica Chimica Acta*.

[21] Cheruvanky A., Zhou H., Pisitkun T. (2007). Rapid isolation of urinary exosomal biomarkers using a nanomembrane ultrafiltration concentrator. *American Journal of Physiology—Renal Physiology*.

[22] Thongboonkerd V. (2011). Study of diabetic nephropathy in the proteomic era. *Diabetes and the Kidney*.

[23] Pisitkun T., Shen R.-F., Knepper M. A. (2004). Identification and proteomic profiling of exosomes in human urine. *Proceedings of the National Academy of Sciences of the United States of America*.

[24] Conde-Vancells J., Rodriguez-Suarez E., Gonzalez E. (2010). Candidate biomarkers in exosome-like vesicles purified from rat and mouse urine samples. *Proteomics—Clinical Applications*.

[25] Ardoin S. P., Shanahan J. C., Pisetsky D. S. (2007). The role of microparticles in inflammation and thrombosis. *Scandinavian Journal of Immunology*.

[26] Qu Y., Du E., Zhang Y., Li S., Han R., Qiu M. (2012). Changes in the expression of bone morphogenetic protein 7 and tamm—horsfall protein in the early stages of diabetic nephropathy. *Nephro-Urology Monthly*.

[27] Agardh C.-D., Stenram U., Torffvit O., Agardh E. (2002). Effects of inhibition of glycation and oxidative stress on the development of diabetic nephropathy in rats. *Journal of Diabetes and its Complications*.

[28] Bhensdadia N. M., Hunt K. J., Lopes-Virella M. F. (2013). Urine haptoglobin levels predict early renal functional decline in patients with type 2 diabetes. *Kidney International*.

[29] Torffvit O., Agardh C.-D., Thulin T. (1999). A study of Tamm-Horsfall protein excretion in hypertensive patients and type 1 diabetic patients. *Scandinavian Journal of Urology and Nephrology*.

[30] Matheson A., Willcox M. D. P., Flanagan J., Walsh B. J. (2010). Urinary biomarkers involved in type 2 diabetes: a review. *Diabetes/Metabolism Research and Reviews*.

